# Sociodemographic Factors and Awareness of Consanguineous Marriages in Rural Northern Karnataka: A Cross-Sectional Study

**DOI:** 10.7759/cureus.76376

**Published:** 2024-12-25

**Authors:** Arun P Sasi, Rekha Udgiri, Praveen Ganganahalli, Vijaya M Sorganvi

**Affiliations:** 1 Community Medicine, Shri. B. M. Patil Medical College, Hospital and Research Centre, BLDE (Deemed to be University), Vijayapura, IND

**Keywords:** awareness, consanguineous marraige, northern karnataka, rural area, sociodemographic factors

## Abstract

Background

Consanguineous marriages, defined as unions between closely related individuals, are influenced by a complex interplay of cultural, social, economic, religious, and demographic factors. These marriages are prevalent among communities such as Hindus, Muslims, Jews, Buddhists, Christians, and Parsis in Southern and Western Asia, with significant regional variations within India. There is a lack of appropriate decision-making among women in consanguineous unions, particularly those with a low level of educational attainment, which leads to an increase in the prevalence of consanguineous marriages. This needs to be addressed through more studies and educational campaigns. Therefore, the primary objective of this study is to assess knowledge about the consequences of consanguinity and the sociodemographic factors related to consanguinity.

Methodology

A cross-sectional study was conducted in Unnat Bharat Abhiyan villages (Ukkali, Donur, Yambatnal, Hegadihal, Deginal) enrolled under Shri B. M. Patil Medical College, Hospital and Research Centre, BLDE (Deemed to be University), Vijayapura, India, which was selected using a lottery method. The houses were selected randomly, focusing on ever-married females aged 15-49 within the reproductive age group. Data on sociodemographic profile, marital status, and awareness status of the participants were collected from March 2023 to April 2024 using an interview technique with a pretested, semi-structured questionnaire.

Results

The study involved the enrollment of a total of 108 participants (Donur - 24, Yembatnal - 32, Ukkali - 27, Deginal - 10, Hegadihal - 15). Among the respondents, only 37 participants (34%) know the specific health and genetic consequences of consanguinity, such as increased risk of genetic disorders (stillbirth, milestone delay, cerebral palsy). The study highlights a significant association between respondents' knowledge of the consequences of consanguineous marriage, their literacy level, and socioeconomic status.

Conclusion

The study concludes that literacy levels are inversely related to the prevalence of consanguineous marriages, suggesting that education holds the key to reducing this practice. The results of this study highlight the necessity of tackling this problem through a multifaceted strategy. This strategy should include culturally sensitive educational programs, economic empowerment initiatives for women, and religious discourse that promotes genetic diversity, offering the potential for change and progress.

## Introduction

Consanguineous marriage is defined as a union between two individuals who are related as second cousins or closer. This practice, deeply rooted in cultural and religious traditions, involves unions between more distantly related people and closely related people, such as uncles and nieces, second or third cousins, etc. First-degree cousin marriages are the most predominant form of consanguinity found in most studies. The frequency of consanguineous marriages varies significantly among different populations, influenced by factors related to religion, culture, social status, economy, and geography. This practice is common among Hindus, Muslims, Jews, Buddhists, Christians, and Parsis in parts of southern and western Asia [1].

Data on consanguineous marriage in India are provided by the National Family Health Surveys (NFHS)-4 in 2015-2016 and NFHS-5 in 2019-2021, which indicate an overall prevalence of 9.9% and 11%, respectively. With 28%, Tamil Nadu has the highest rate, followed by 27% in Karnataka. Muslims in North India and Hindus in South India are more likely to engage in consanguineous marriages [2].

As treatment for once-lethal genetic disorders improves and nuclear family patterns and urbanization become more prevalent, there is potential for a decrease in consanguineous marriages in urban areas. The major factors, including cultural, educational, and financial backgrounds, play a significant role in consanguineous marriages. Attainment of increased literacy and financial upliftment also reduces the reluctance towards the follow-up of traditional culture, which may ultimately lead to a decrease in consanguineous marriages. This shift, which signifies a significant cultural change, weighs the advantages of consanguineous marriage in terms of social and financial status against the potential health risks. It offers hope for a healthier future as the population's economic status advances [3].

Enhanced knowledge of personal risk factors and better decision-making are facilitated by increased health literacy, especially regarding health issues, self-care, and disease prevention. Lower educational attainment, early marriage, and early childbearing are common characteristics of women in consanguineous unions. Consanguineous marriages are most commonly documented in rural areas and low-socioeconomic communities. This emphasizes the need for a community education program, a higher standard of education, and greater awareness about the effects of kinship among rural residents [4].

However, the lack of community-based studies focusing on consanguineous marriages in rural areas, particularly in Northern Karnataka, underscores the urgent need for further research and awareness initiatives. There is a significant limitation in the geographical scope of the sample, which needs a longitudinal design in the future to allow researchers to track changes over time, providing a better understanding of the development and progression of the phenomena being studied. This call to action should motivate us to fill these knowledge gaps and initiate more awareness programs. The present study intends to increase awareness among consanguineous marriage participants in rural areas and also study the sociodemographic factors related to consanguineous marriages among the participants.

## Materials and methods

The present cross-sectional study was conducted using a lottery method, and the houses were selected randomly, focusing on ever-married females aged 15-49 years within the reproductive age group. The study was conducted in the households of Unnat Bharat Abhiyan villages, known for their unique sociodemographic characteristics, and enrolled under Shri B. M. Patil Medical College, Hospital and Research Centre, BLDE (Deemed to be University), Vijayapura, India. The study focused on ever-married females between the ages of 15-49 years in the reproductive age group residing in these villages from March 2023 to April 2024.

A comprehensive interview technique was employed in the study, using a pretested, semi-structured questionnaire (Appendix, Table 9) to ensure a thorough understanding of the participants' perspectives. This questionnaire covered various sociodemographic aspects, including name, age, occupation, educational status, religious affiliation, average monthly income, family type, and marital status. The socioeconomic status of the participants was assessed using the Modified B.G. Prasad classification.

After obtaining ethical clearance on August 26, 2022, from Shri B. M. Patil Medical College, Hospital and Research Centre (BLDE (DU)/IEC/693/2022-23), questionnaires were prepared in English, translated into Kannada, and administered following a pilot study in 30 households.

The study’s purpose was explained to participants, and written informed consent was obtained. Data collection involved a pretested, semi-structured questionnaire, with a thorough enumeration of all households in Unnat Bharath Abhiyan villages (Yambatnal, Donnur, Haggadihal, Deginal, Ukkali), conducted to enlist all married women within the reproductive age group. Subsequently, consanguineous marriages were identified, and the history of consanguineous marriages was gathered through door-to-door visits. The study included all ever-married women in the reproductive age group of 15-49 years and permanent residents of the area, excluding those who were not married or not willing to give consent for the study.

Statistical analysis was conducted, and data obtained were entered into a Microsoft Excel sheet (Microsoft® Corp., Redmond, WA, USA). The analysis was performed using IBM SPSS Statistics for Windows, Version 24 (Released 2016; IBM Corp., Armonk, NY, USA). The association between categorical variables was computed using the Chi-square test. A p-value of 0.05 was considered statistically significant.

## Results

This study shows that 35 participants (32.4%) belong to the age group of 24-29 years, followed by 24 participants (22.2%) in the age group of 30-34 years, whereas 21 participants (19.4%) are in the age group of 19-23 years. There are 0.9% fewer people under 18 years of age, and no participants are in the 46-49 age group.

Among our study participants, 94 were Hindus (87%), who were predominant, and 14 (13%) were Muslims. Education levels of the participants vary, with 41.7% having completed high school, 25% having attended primary school, 12% having completed pre-university courses (PUCs), 11.1% being graduates, and 10.2% being uneducated. Regarding spouses' education, 38.9% have completed high school, 23.1% are graduates, 16.7% were uneducated, followed by 13.9% having completed PUC, and 7.4% having attended primary school.

In the present study, 96.3% of the participants are homemakers, with a small fraction engaged in agriculture (0.9%), services (1.9%), and labor (0.9%) by occupation. The spouse occupations predominantly include agriculture (42.6%), labor (25.9%), services (13.9%), daily wages/others (15.7%), and business (1.9%). Family structures show that 58.3% live in three-generation families, 29.6% in nuclear families, and 12% in joint families (Table 1).

**Table 1:** Distrribution of sociodemogrpahic correlates of consanguineous marriage Nuclear Family: When the family unit consists of a husband, wife, and children, it is called a nuclear family. Joint Family: This family can be considered a lateral extension of the nuclear family. It consists of nuclear families of siblings (brothers in the patrilocal system and sisters in the matrilocal system), and the eldest brother/sister has the position of authority. Three-Generation Family: This is similar to a joint family, but the reason for a married son living with the parents is economical, not social. PUC: Pre-university course

	Variables	Frequency	Percentage (%)
Age	<18	1	0.9
	19-23	21	19.4
	24-29	35	32.4
	30-34	24	22.2
	35-39	20	18.5
	40-45	7	6.5
	46-49	0	0
Religion	Hindu	94	87
	Muslim	14	13
Participants education status	Uneducated	11	10.2
	Primary school	27	25
	High school	45	41.7
	PUC	13	12
	Graduate	12	11.1
Spouse educational status	Uneducated	18	16.7
	Primary school	8	7.4
	High school	42	38.9
	PUC	15	13.9
	Graduates	25	23.1
Participant’s occupation	Homemaker	104	96.3
	Agriculture	1	0.9
	Services	2	1.9
	Labor	1	0.9
Spouse occupational status	Agriculture	46	42.6
	Labor	28	25.9
	Services	15	13.9
	Daily wages	17	15.7
	Business	2	1.9
Type of family	Nuclear	32	29.6
	Joint family	13	12
	Three-generation	63	58.3
Total		108	100

**Figure 1:**
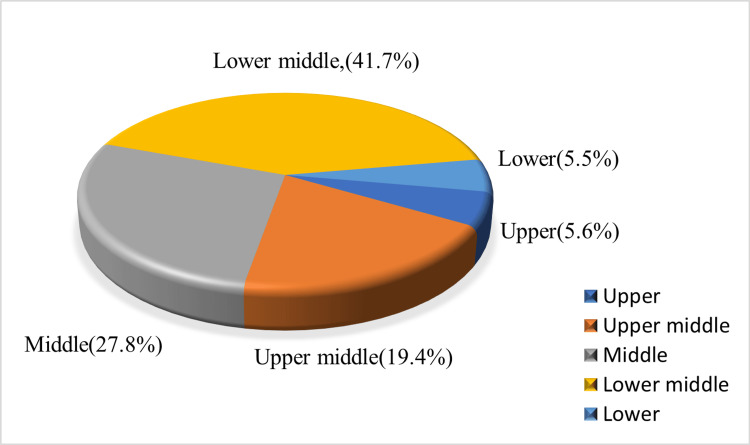
Distribution of the socioeconomic status among the study participants using modified B.G. Prasad classification

Among the participants, with regard to socioeconomic status, 45% belong to the lower middle class, followed by 30% in the middle class, 19.4% in the upper middle class, and 5.6% belong to both upper and lower socioeconomic status according to the modified B.G. Prasad classification (Figure 1).

In our study, among 108 married females, 3.7% were widowed, and 0.9% lived separated from their husbands. Regarding the duration of the marriage, the majority of the participants had a married life of more than 10 years (55.6%), followed by one to three years (17.6%) and four to six years (12%). Among the study participants, the majority were married between the ages of 18-20 years (57.4%), followed by 21-22 years (28.7%) and 17 years (9.3%). Most participants had their first child at the age of 20 years (63%), followed by 21-23 years (25.9%). The maximum number of participants had up to two children (64.8%); 26.9% had three to four children, and 7.4% had no children (Table 2).

**Table 2:** Distribution of marital and reproductive status among the consanguineously married study participants NA: Those who do not have children

Parameters	Variables	Frequency	Percentage (%)
Duration of marriage	1-3	19	17.6
	4-6	13	12.0
	7-9	16	14.8
	>10	60	55.6
Age at marriage	<17	10	9.3
	18-20	62	57.4
	21-22	31	28.7
	23-25	3	2.8
	>26	2	1.8
Age of first delivery	≤20	68	63.0
	21-23	28	25.9
	24-27	4	3.7
	N/A	8	7.4
Number of children	≤2	70	64.8
	3-4	30	26.9
	N/A	8	7.4
Total		100	100

**Figure 2:**
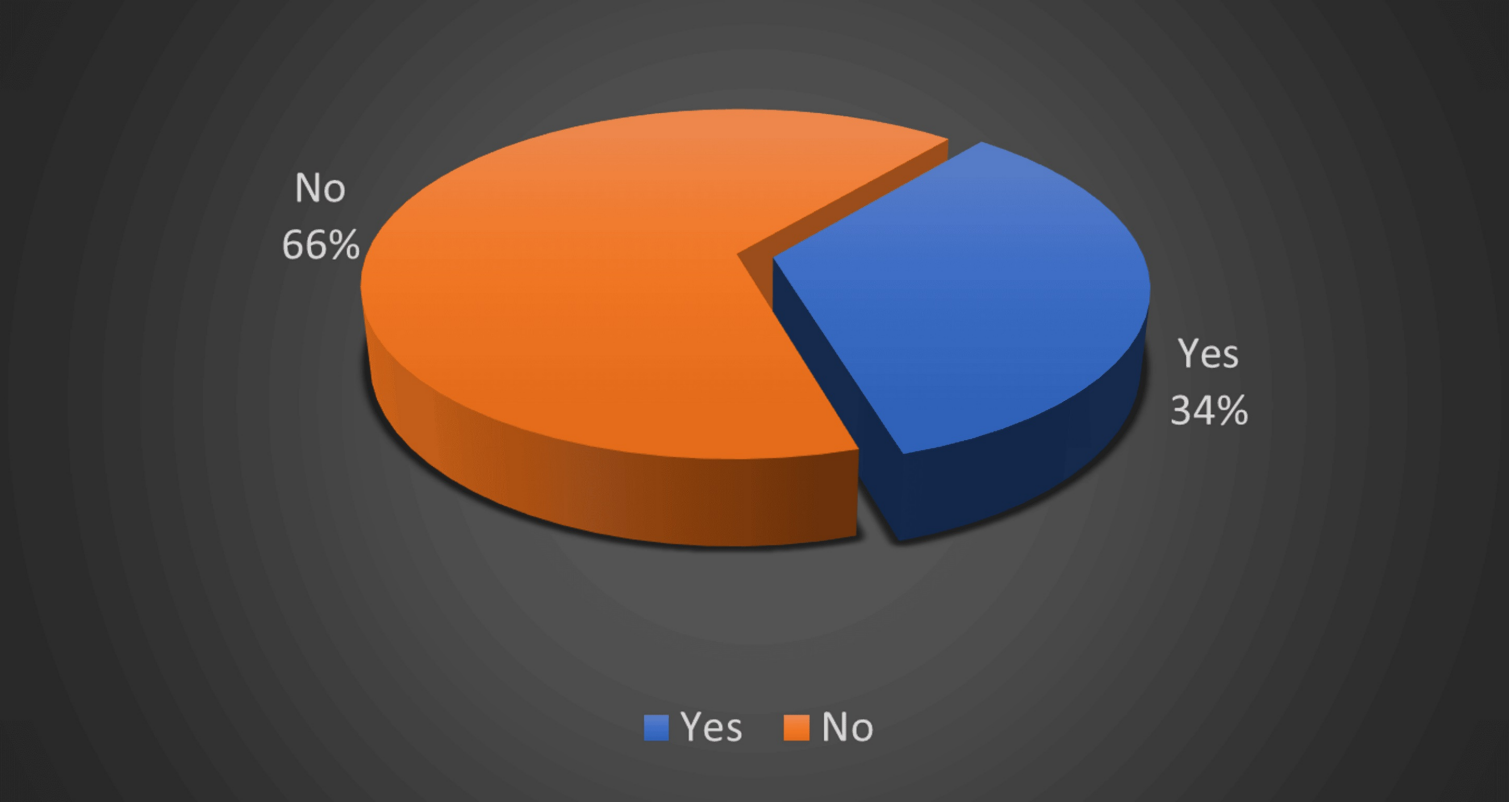
Distribution of knowledge regarding the consequences of consanguineous marriages among the study participants

It is alarming to know that in the present study, only 34% of the participants had knowledge about the consequences of consanguineous marriage (Figure 2).

**Figure 3:**
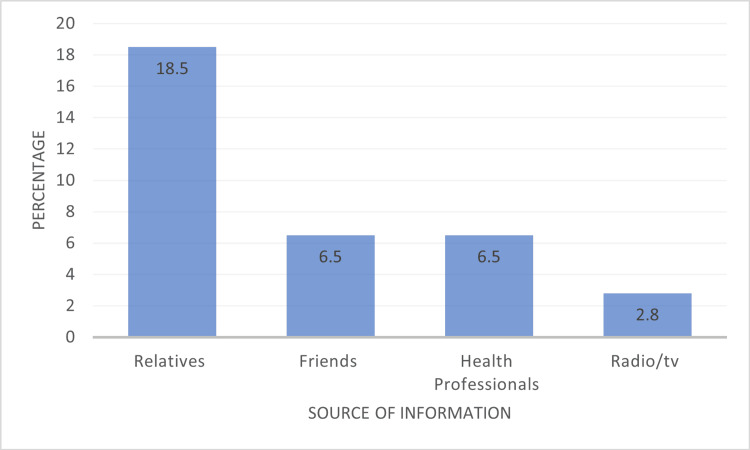
Distributiion of information source about the consequences of consanguineous marriage among the study paricipants

Among the study participants, 18.5% gained knowledge regarding the consequences of consanguineous marriage from relatives, followed by friends (6.5%), health professionals (6.5%), and 2.8% who gained knowledge through mass media communications like television and radio (Figure 3).

**Figure 4:**
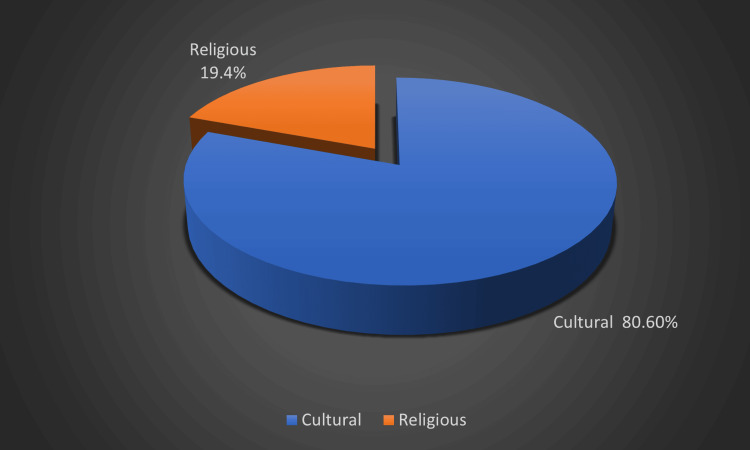
Distribution of reason for consanguinity among participants

The reasons for consanguinity in our study were due to cultural (80.6%) and religious factors (19.4%), which are mainly attributed to factors like preserving wealth and property within families, strengthening familial ties and social cohesion, and religious factors like encouraging cousin marriages (Figure 4).

In our study, we observed that the majority of the participants in the age group 24-29 (32.4%) had more knowledge about the consequences of consanguineous marriage, followed by those in the 35-39 age group (24.3%). Overall, participants older than 24 years had knowledge about consanguineous marriage (85%) (Table 3).

**Table 3:** Distribution of participant's knowledge on consequences of consanguineous marriage among the different age groups n denotes the variable.

Participant's knowledge	Yes		No		Total
Age	n	(%)	n	(%)	n
≤18	0	0	1	1.4	1
19-23 years	5	13.5	16	22.5	21
24-29 years	12	32.4	23	32.3	35
30-34 years	6	16.2	18	25.3	24
35-39 years	9	24.3	11	15.4	20
40-45 years	5	13.5	2	2.81	7
Total	37	100	71	100	108

The study highlights a significant association between the participant's educational status and knowledge of the consequences (developmental delays, handicaps) of consanguineous marriage, with p = 0.000 (Table 4).

**Table 4:** Association of the participant's knowledge on consequences of the consanguineous marriage with respect to participant's educational status n denotes the variable, with statistical analysis performed using the Chi-square test. *p-value < 0.05 indicates statistical significance. PUC: Pre-university course

Participant's knowledge	Yes		No		Total	Chi-square	p-value
Educational status	n	(%)	n	(%)	n	29.67	0.000*
Uneducated	2	18.2	9	81.8	11		
Primary school	5	18.5	22	81.5	27		
High school	19	42.2	26	57.8	45		
PUC	1	7.7	12	92.3	13		
Graduate	10	83.3	2	16.7	12		
Total	37	34.2	71	65.7	108		

Similarly, spouse education also shows a statistically significant relationship between the participant's knowledge of the consequences of consanguineous marriage and the spouse's educational status, with p = 0.005 (Table 5).

**Table 5:** Association between particpant's knowledge of consequences on consanguineous marriage with respect to spouse’s educational status n denotes the variable, with statistical analysis performed using the Chi-square test. *p-value < 0.05 indicates statistical significance. PUC: Pre-university course

Participant's knowledge	Yes		No		Total	Chi-square	p-value
Spouse's educational status	n	(%)	n	(%)	n	14.6	0.005*
Uneducated	8	44.4	10	55.6	18		
Primary school	5	62.5	3	37.5	8		
High school	19	45.2	23	54.8	42		
PUC	1	6.7	14	93.3	15		
Graduate	4	16.0	21	84.0	25		
Total	37	34.2	71	65.7	108		

The study highlights that the majority of our study participants were homemakers (35.6%). A significant association was found between the participant's occupation and the participant's knowledge of the consequences of consanguineous marriage, with p = 0.0539 (Table 6).

**Table 6:** Association between the participant's knowledge of consequences on consanguineous marriage in relation with respect to the participant's occupation n denotes the variable, with statistical analysis performed using the Chi-square test. *p-value < 0.05 indicates statistical significance.

Participant's knowledge	Yes		No		Total	Chi-square	p-value
Occupation	n	(%)	n	(%)	n	2.165	0.0539*
Homemaker	37	35.6	67	64.4	104		
Agriculture	0	0.00	1	100	1		
Services	0	0.00	2	100	2		
Labor	0	0.00	1	100	1		
Total	37	34.2	71	65.7	108		

It is good to know that all the spouses were involved in one job or another. The present study highlights a significant association between the participant's knowledge of the consequences of consanguineous marriage and their spouse's occupation, with p = 0.018 (Table 7).

**Table 7:** Association between participant's knowledge of consequences on consanguineous marriage with respect to spouse’s occupational status n denotes the variable, with statistical analysis performed using the Chi-square test. *p-value < 0.05 indicates statistical significance.

Participant's knowledge	Yes		No		Total	Chi-square	p-value
Spouse's occupation	n	(%)	n	(%)	n	11.92	0.018*
Labor	9	32.1	19	67.9	28		
Agriculture	21	45.7	25	54.3	46		
Services	0	0.00	15	100	15		
Daily wages	7	41.2	10	58.8	17		
Business	0	0.00	2	100	2		
Total	37	34.2	71	65.7	108		

It is surprising to know that lower-middle-class participants have more knowledge regarding the consequences of consanguineous marriage (56.8%), followed by the upper-middle class (21.6%). There is no significant relationship between socioeconomic status and the participant's knowledge of the consequences of consanguineous marriage (Table 8).

**Table 8:** Association between the participant's knowledge with respect to participant's socioeconomic status according to modified B.G. Prasad classification n denotes the variable, with statistical analysis performed using the Chi-square test. A p-value > 0.05 indicates no statistical significance.

Participant's knowledge	Yes		No		Total	Chi-square	p-value
Socioeconomic status	n	(%)	n	(%)	n	8.76	0.68
Upper	1	2.7	5	7.0	6		
Upper middle	8	21.6	13	18.3	21		
Middle	7	18.9	23	32.4	30		
Lower middle	21	56.8	24	33.8	45		
Lower	0	0.00	6	8.5	6		
Total	37	34.2	71	65.7	108		

## Discussion

Our study observed that 10% of the participants were illiterate. Similarly, the educational status of spouses follows a similar trend, with 16.7% being illiterate. The degree of education of the participants and their awareness of consanguineous marriages are significantly correlated. Interestingly, 34.3% of study participants are aware of the drawbacks of consanguineous marriage.

Saho et al. conducted a community-based cross-sectional study based on the NFHS-4 round of data. Consanguineous marriages were more predominant among participants with no education compared to those with secondary or higher education [5]. Another descriptive-analytical study conducted by Sedehi et al. in Iran observed a significant association between the participants' knowledge and education [6].

Another study by El Goundali et al. conducted a systematic review showing that, in Oman, women’s education levels showed no significant association with inbreeding when the effects of other factors were adjusted. Similarly, in Sana’a and Riyadh, education and inbreeding showed no correlation. This contradicts the findings observed in our study [7].

Another cross-sectional descriptive study by Khan and Mazhar observed a significant association between educational status and preference for consanguineous marriage. The findings they observed in their study are similar to those we observed in our study [8]. Another study conducted by Akrami et al. observed a significant relationship between education and awareness about consanguineous marriage. Participants with higher education were more reluctant toward consanguineous marriage. The findings they observed in their study are similar to those we observed in our study [9].

These studies show that literacy plays a vital role in reducing consanguineous marriage, as it helps individuals make informed decisions and modulates their attitude and practices toward consanguineous marriage.

Our study observed that 96.3% of the participants were homemakers. An association was found between the participants' occupation and their knowledge of the consequences of consanguineous marriage. Similarly, a significant association was found between the spouse’s occupation and the knowledge of the consequences of consanguineous marriage. Our study also observed that 42.6% of spouses were involved in agriculture. Additionally, the study found that, out of the total participants, 64.4% did not know the consequences of consanguineous marriage.

A cross-sectional study conducted by Joseph et al. in Mangalore, with 178 respondents, observed that most of their participants were housewives (63.1%), but no association was found in their research. The findings they observed in their study are similar to those of our research [10]. This could be because homemakers often prioritize family stability, cultural tradition, and economic security, which leads to a higher priority for consanguineous marriage. Regarding spouses, as our study area is rural, more families are involved in labor, daily wages, and agriculture. Their potential marriage partners are limited, and the family prefers known and trusted individuals; this can lead to a higher incidence of consanguineous marriage.

A cross-sectional epidemiological study conducted by Riaz et al. found that consanguineous marriages were higher among subjects involved in agriculture/farming. On the other hand, kinship was lowest among husbands working in offices, services, and those engaged in businesses. The findings they observed in their study are similar to those we observed in our study [11].

Another community-based cross-sectional survey was conducted by Jabeen and Malik in the Bhimbri district of Jammu and Kashmir. In their research, they observed that no significant association was found between consanguineous marriage and spouse occupation. In their study, spouses' occupations were divided into skilled and non-skilled categories; 63% were professionals, and 63% of the husbands worked abroad. The findings they observed in their study contradict those we observed in our study [12]. The discrepancies in their findings may be due to greater exposure to the outer environment and increased interaction in workplaces, which helped them gather more information about consanguineous marriage.

In our study, most participants belong to the lower-middle class (41.7%), and surprisingly, there is more knowledge of the consequences of consanguineous marriage among those of lower-middle socioeconomic status (41.7%). Abbas et al. conducted a cross-sectional analytical study in Pakistan with 254 respondents and observed in their research that most of the participants in their study belonged to a lower socioeconomic status [13]. 

Kamal et al. conducted a cross-sectional study in Pakistan and observed that most consanguineous marriages occur in lower socioeconomic status groups. Similarly, another study conducted by Jurdi and Saxena observed in their research that couples living in nuclear households and those of middle to high economic status are less likely to be in consanguineous marriages, regardless of their residence. This finding observed in their study contradicts the findings we observed in our study [14,15]. These study findings align with those observed in our study. The reason could be that there is no need to pay a dowry if the marriage is within the family, which may be linked to the desire to preserve ancestral property without marrying outside the family.

The present study promotes developing and supporting education programs that raise awareness about the consequences of consanguineous marriage and also provides genetic counseling in health and wellness centers.

## Conclusions

Our study concluded that literacy levels were inversely related to the prevalence of consanguineous marriages, suggesting that education may play a pivotal role in reducing these practices. Education enables individuals to make appropriate decisions and provides good knowledge, attitudes, and practices in the community. Since there is a lack of proper knowledge about the consequences of consanguineous marriage, these marriages continue to occur. The lower level of educational status is found to be the primary barrier to providing awareness to the community regarding the consequences of consanguineous marriage.

The present study recommends that there is a major need for educating and counseling the participants and their family members regarding the consequences of consanguineous marriage. Overall, it shows that consanguineous marriage requires a multifactorial approach, incorporating educational, economic, and cultural factors. It is urgent that we recommend, develop, and support educational programs that raise awareness of the consequences of consanguineous marriage.

## Appendices

**Table 9:** Semistructured questionnaire used in the present study

Parameter	Response
Age	…………….
Religion	Hindu/Muslim/Christian/Others/Specify
Educational status	SSLC/PUC/Graduate/Postgraduate
Occupation	Homemakers/Services/Labor/Business/Agriculture/Others
Husband’s educational status	SSLC/PUC/Graduate/Postgraduate
Husband’s occupation	Services/Labor/Business/Agriculture/Others/Daily wages
Number of family members	…………….
Relation to women	Mother/Sister/Brother/Son/Husband/Father-in-law/Mother-in-law/Others .……………
Knowledge regarding family planning methods	Yes/No
If yes, which methods	…………
Have you adopted any family planning method	Yes/No
If yes, specify	………….
Income per month	…………….
Per capita income	………………
Socio-economic status	Upper/Upper middle/Middle/Lower middle/Lower
Duration of marriage	…………..
Age at marriage	…………….
Age at first delivery	………………
Number of children	……………..
Age of children	……………..
Are you aware of the consanguineous marriage	Yes/No
If yes, how	Newspaper/Radio/TV/Internet/Relatives/Friends/Health professionals/Others
Reason for consanguinity	Open-ended responses ……………/Cultural/No dowry/Poverty/Economical/Religious/Any others
